# Interference Phenomena and Stimulated Emission in ZnO Films on Sapphire

**DOI:** 10.3390/ma15186409

**Published:** 2022-09-15

**Authors:** Arsen E. Muslimov, Andrey P. Tarasov, Vladimir M. Kanevsky

**Affiliations:** Federal Scientific Research Centre “Crystallography and Photonics” of Russian Academy of Sciences, Shubnikov Institute of Crystallography, 119333 Moscow, Russia

**Keywords:** zinc oxide, sensors, films, sapphire, gold buffer layer, microcrystals, interference, stimulated emission, refractive index sensors, UV luminescence

## Abstract

We studied the texturing, roughness, and morphology features of ZnO films grown on the R (1$\bar{1}$02)-, M (10$\bar{1}$0)-, A (11$\bar{2}$0)-, and C (0001)-planes of sapphire, as well as their optical and luminescent properties. We showed that the growth conditions, substrate orientation, and the presence of a buffer layer significantly affected the structure and morphology of the growing films, which was reflected in their optical and radiative properties. In particular, films grown on the A- and M- planes of sapphire showed the highest UV radiation brightness values and exhibited stimulated emissions upon pulsed photoexcitation. The dependence of the topography of the film surface on the substrate orientation allowed the formation of a smooth continuous film with pronounced interference properties using the R- and M- planes of sapphire. A change in the crystallographic orientation, as well as a significant enhancement in crystallinity and luminescence, were observed for ZnO films grown on R-plane sapphire substrates with a gold buffer layer as compared to films grown on bare substrates. At the same time, the use of gold facilitates a significant smoothing of the film’s surface, retaining its interference properties. The sensitivity of interference and laser properties to changes in the external environment, as well as the ease of fabrication of such structures, create prospects for their application as key elements of optical converters, chemical and biological sensors, and sources of coherent radiation.

## 1. Introduction

Semiconductor electronics based on planar technologies led to the rapid development of the industry in the second half of the last century [1]. The breakthrough in modern technologies is mainly associated with the unique properties of nanoobjects [2]. With all the advantages of nanoobjects, there is a technological complexity involved in their integration into multi-level systems of a larger scale, for example, sensor devices. For this reason, planar structures remain indispensable in many areas of modern technology [3]. Zinc oxide (ZnO), due to its unique combination of electrophysical, optical, piezoelectric properties, remains one of the most studied film materials [4].

In biodiagnostics, it has been proposed to implement ZnO-based sensors that use an amplification of the fluorescence and the electrochemical responses from biological objects [5]. Previously, ZnO films were used in “lab-on-a-chip” technology and biosensors based on acoustic waves [6]. Furthermore, the development of gas sensors on ZnO films that use various physical effects for sensing is also topical [7,8,9].

Another classical area of application of ZnO film structures is the development of light-emitting devices operating in the near-UV and violet spectral ranges [10,11,12], including optical amplifiers and generators [13,14,15]. In this context, among the advantages of ZnO are their high exciton binding energy (60 meV) and refractive index, their radiation resistance, and their low manufacturing cost [16,17]. Moreover, ZnO has become a model material for studying excitonic processes in semiconductors. In addition, due to the high sensitivity of the physical properties of micro/nano-optical resonators and lasers to changes near their surface, laser effects in ZnO can also be used in sensing. In particular, whispering gallery mode (WGM) lasing in ZnO microrods and microwires can be utilized for stress [18], biological [19], gas [20], and temperature [21] sensing.

Despite the wide range of potential applications, film structures are mainly introduced into individual unique devices and this is mainly due to the great influence of the synthesis conditions on the properties of the films and their low reproducibility. In addition, there is a direct dependence of a film’s properties on its thickness, orientation, and substrate material. All this imposes high requirements on the technology used for the fabrication of ZnO thin films. In this regard, it is very important to develop new directions for the use of ZnO films and methods which are less dependent on the structural-phase composition, synthesis parameters, and material features.

In [22], the authors proposed the use of the luminescent properties of nanoporous aluminum oxide as markers for biological systems. The idea is to represent the set of oscillations observed in the luminescence spectrum in the form of a barcode in which the bar width corresponds to the oscillation line intensity. However, the fabrication of nanoporous ZnO with high regularity involves some technological difficulties. In [23], another approach based on using the features of the luminescent properties of ZnO films grown on the rhombohedral plane of sapphire with a gold buffer layer was proposed. In the luminescence spectra of such structures, a set of oscillations was found, which is a consequence of classical film interference. By analogy with porous aluminum oxide, a set of oscillations can be represented as a barcode.

Another example of a possible application of ZnO film structures that does not require high crystallinity and stoichiometry are refractive index sensors. The most sensitive sensors used today are based on fiber optics, waveguides, ring resonators, and plasmonic structures, and often use sensitive spectrometers and laser radiation [24]. The planar structure of a ‘smooth ZnO film/sapphire substrate’, using a simple LED emitter as a light source and a photodiode for registration, can serve as a prototype of the simplest refractive index sensor. Such a sensor, in particular, can be useful for in situ studies of the photodegradation of optically dense media, such as glucose or sucrose, without the use of labor-intensive spectrophotometric techniques. The resolution of such a sensor depends on the thickness of the ZnO film and the roughness of its surface, which, in turn, is determined by the orientation of the film, the degree and directions of texturing, and the synthesis parameters in general.

ZnO films traditionally exhibit texturing along the [0001], [10$\bar{1}$0], and [11$\bar{2}$0] directions [25,26,27]. The density of surface free energy calculated in [28] for the (0001), (10$\bar{1}$0), and (11$\bar{2}$0) planes was 0.099, 0.209, and 0.123 eV/Å^2^, respectively. Due to the fact that they have the lowest value for the (0001) plane, ZnO films are characterized by [0001]-texturing. As a consequence, (0001)-oriented ZnO films are formed even on nonorienting substrates. The greatest structural-geometric similarity of the ZnO and sapphire lattices has been observed for the A (11$\bar{2}$0) sapphire plane, whereas for the C (0001) sapphire plane, the least similarity has been observed. In both cases, [0001]-textured films are formed. A feature of the surface morphology of [0001]-textured films is that growing crystallites form a rough surface. To smooth this surface, a porous buffer sublayer is required, which reduces the density of ZnO seeds and restrains the growth of each nucleus at the expense of neighboring ones. As a result, the crystallites grow at approximately the same rate. Gold can be used for this sublayer. The mechanism of the catalytic synthesis of an ensemble of [0001]-oriented ZnO whisker microcrystals using gold is known [29,30]. According to the calculation results from [28], the free energy densities for the (0001) and (11$\bar{2}$0) ZnO planes are close in value, and with an increase in the growth rate, the [11$\bar{2}$0] texture quite easily transforms into isolated [0001] ZnO microcrystals. These features make it possible to form bitextured ZnO films with the main [11$\bar{2}$0] texture and an ensemble of [0001]-oriented microcrystals.

In accordance with the structural and geometric similarity of the film and substrate lattices, for the synthesis of [11$\bar{2}$0]-oriented ZnO films with their basal axis lying in the plane of the substrate, the use of the rhombohedral R (1$\bar{1}$02) sapphire plane is suitable [27,31]. In this case, the lattice mismatch remains 1.53% along the [0001] direction of ZnO. As we found previously [32], [10$\bar{1}$3] ZnO films are formed on the M (10$\bar{1}$0)-plane of sapphire.

This brief analysis demonstrates the possibility of controlling the roughness, texturing, and morphology of ZnO films using the parameters of the synthesis process and the orientation of sapphire substrates.

In this work, we studied the effect of texturing, roughness, and morphology features of ZnO films grown on the R (1$\bar{1}$02)-, M (10$\bar{1}$0)-, A (11$\bar{2}$0)-, and C (0001)-planes of sapphire, assessing their optical, interference, and laser characteristics.

## 2. Materials and Methods

The R (1$\bar{1}$02), M (10$\bar{1}$0), A (11$\bar{2}$0), and C (0001) sapphire planes were used to grow ZnO films. Chemical-mechanical polishing was applied to both sides of the sapphire substrates to obtain a roughness of about 0.2 nm according to the technique described in [33,34]. Four samples of ZnO films were obtained on sapphire substrates of each type. Samples are further designated as types R, A, M, and C. Films of type R were considered in two versions: without a buffer layer (type R-I) and with a buffer porous gold layer (type R-II). A porous layer of gold (thickness about 200 nm) was formed via thermal deposition, after which it was heated at a temperature of 730 °C for 30 min. The thickness of a gold layer was chosen in such a way as to provide the possibility of measurements both in reflected and transmitted light.

The films were synthesized via magnetron sputtering (discharge current, 100 mA) at a temperature of 810 °C in an oxygen atmosphere at a pressure of 1.33 Pa. The synthesis time was 25 min. The film growth rate was ~2 nm/s. All types of ZnO films were synthesized under identical conditions. The optimum growth rate of thin films was 2 nm/s. Since the growth rate for (0001) ZnO is the highest and, for comparison, it was desirable to obtain type R-I and R-II films with the same thickness of ~3 µm, the deposition time was set to 35 min for R-I and 25 min for R-II. Since we intended to study the effect of the film thickness on their optical and interference properties, as well as to find out the conditions for the appearance of laser effects, the films of types A and M were deposited for 90 min. The stoichiometric ZnO ceramic target was used in the synthesis process. To increase the chemical stability of the film surface, coating with an aluminum layer about 10 nm thick with further annealing in air at 700 °C was performed.

Structural studies of the films were performed using reflection electron diffraction (horizontal electron diffraction; accelerating voltage is 75 kV) and X-ray diffraction (XRD). XRD patterns were obtained on a PANalytical Empyrean diffractometer in the Bragg–Brentano geometry. Radiation from a copper anode (CuK_α2_ = 1.54 Å) was used. XRD patterns were analyzed and reflection peaks were identified using the High Score Plus program, using the ICSD database (PDF-2).

Microscopic studies of the samples were carried out via scanning electron microscopy (SEM) and atomic force microscopy (AFM) using Jeol Neoscope 2 (JCM-6000) and Ntegra Prima microscopes, respectively.

The photoluminescence (PL) of the samples was observed under excitation with the 3rd harmonics (355 nm) of a Q-switched Nd:YAG laser. The pulse duration and repetition rate were ~10 ns and 15 Hz. The size of an excitation spot on the samples was ~200 μm. Emissions of the samples were registered with the use of a Peltier-cooled charge-coupled device camera placed behind the exit slit of a monochromator. To obtain a better signal-to-noise ratio, we applied the averaging of spectra using pulse accumulation.

The spectra of directional reflectance (at an angle of 25°) of the samples were measured using a Solar PB2201 UV-Vis spectrophotometer. The transmittance spectra of the samples were measured in the direction normal to their surface using a Solar PB2201 and a Varian Cary 5000 UV-Vis-NIR spectrophotometer.

All measurements were performed at room temperature.

## 3. Results

### 3.1. Study of the Texturing Processes of ZnO Films

**Figure 1:**
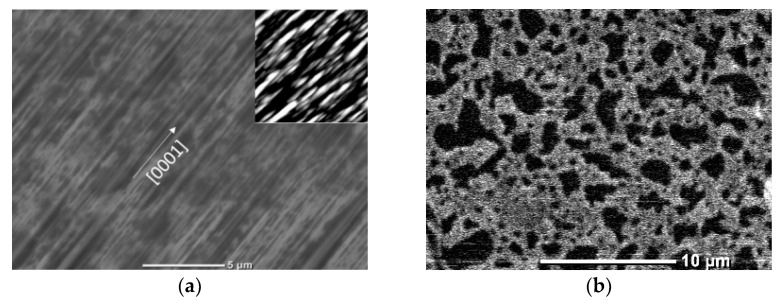

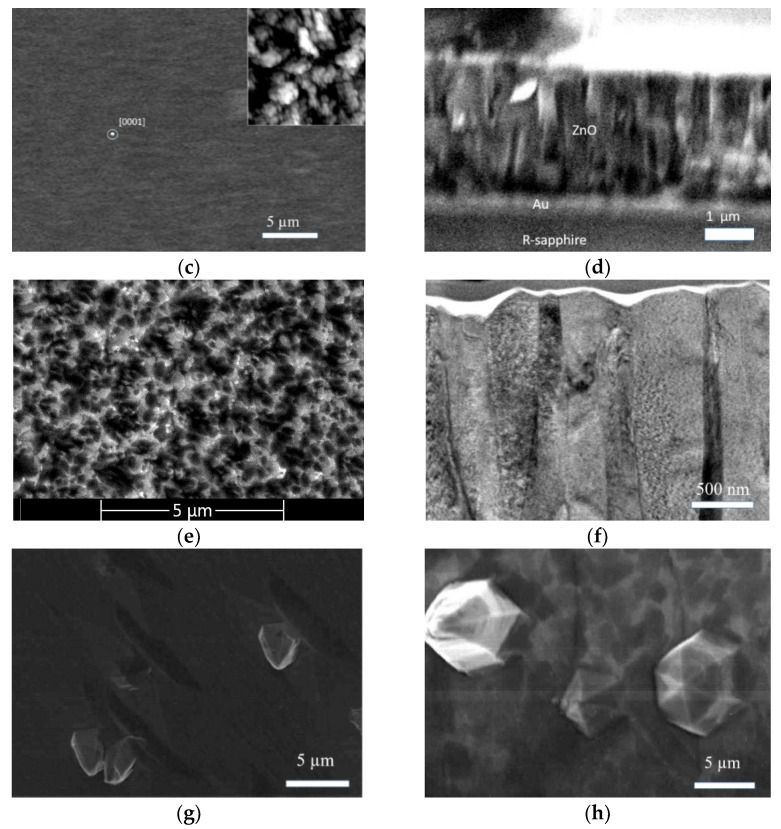
SEM images of ZnO films: (**a**) type R-I film surface; (**b**) porous Au film on sapphire after annealing at 730 °C; (**c**) type R-II film surface; (**d**) type R-II film cross section; (**e**) type C film surface; (**f**) cross section of type C film; (**g**) type A film surface; (**h**) type M film surface. Insets show corresponding AFM images 7 × 7 µm^2^.

In the process of R-I-type film deposition, according to the SEM and AFM data (Figure 1a), a continuous film with a pronounced anisotropic surface was formed. The anisotropy axis coincided with the [0001] axis of ZnO. The roughness was calculated as following. Each point of the surface in the plane (*XY*) was assigned the value of the height *Z* (*XY*) relative to the middle plane, determined by the condition:

(1) $$\frac{1}{S}\underset{S}{\iint }Z\left(XY\right)dx\;dy=\langle Z\rangle =0,$$

where *S* is the area of the surface under consideration and angle brackets denote surface averaging. Then, the root mean square (RMS) deviation from the midline of the surface profile (RMS roughness height *Rq*) can be given by:

(2) $$R_{q}{}^{2}=\frac{1}{S}\underset{S}{\iint }Z^{2}\left(XY\right)dx\;dy=\langle Z^{2}\rangle .$$

The value of *Rq* calculated according to the AFM data and Equations (1) and (2) was 12 nm. The height difference of the relief was up to 47 nm. The average crystallite size can be estimated using the Scherrer equation:

(3) $$D=\frac{K\lambda }{\beta \cos\left(\theta \right)},$$

where *D* is the estimated crystalline size of diffraction planes, *β* is the corresponding fullwidth half maximum of diffraction planes, *k* is constant, and *λ* is the wavelength. The crystallite size was minimal (Table 1), which indicated a large intergranular surface. In addition, the *a* parameter was higher compared to the case of the Standard JCPDS pattern for ZnO (file no: 043-0002). This was probably due to the presence of interstitial zinc. Positively charged zinc ions can flow down to a negatively charged sapphire dielectric substrate in the magnetron discharge plasma. The excess concentration of zinc was especially pronounced in the lower layers of the growing film.

**Figure 2:**
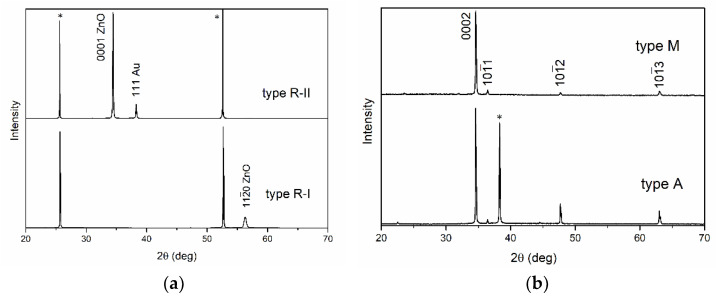
XRD patterns of ZnO films: (**a**) type R-I (bottom), type R-II (top); (**b**) type A (bottom), type M (top). Designation * denotes reflections of the sapphire substrate.

According to the XRD data (Figure 2a), a (11$\bar{2}$0) ZnO film was formed on the R-plane of sapphire during growth. Low roughness was achieved due to the atomically smooth faces of ZnO crystallites located along the [0001] axis parallel to the substrate plane. The ZnO film was formed epitaxially with the fulfillment of the following orientation relations: (11$\bar{2}$0) ZnO ‖ (1$\bar{1}$02) Al_2_O_3_; [1$\bar{1}$00] ZnO ‖ [11$\bar{2}$0] Al_2_O_3_; [0001] ZnO ‖ [1$\bar{1}$0$\bar{1}$] Al_2_O_3_.

During preliminary annealing, a porous Au film was formed (Figure 1b) with lateral pore sizes up to 3 μm. According to XRD data (Figure 2), a (111)-textured Au film was formed. Epitaxy of Au on sapphire substrates refers to the case of weak adhesion of the crystal on the substrate, and epitaxial growth occurs through the formation of three-dimensional clusters. For the case of weak adhesion, a multiplicity of epitaxial relationships is typical, and the parallelism of planes and directions with the highest density of atoms in both lattices is essential. In crystals with a face-centered cubic structure, such as Au, the plane which is closest to the center of the crystal and characterized by the highest atomic density has the highest growth rate. Therefore, a (111)-textured Au film was formed. Au atoms were densest along the <1$\bar{1}$0> direction family. The distance between atoms of the <1$\bar{1}$0> direction was a multiple of 2.83 Å. The arrangement of atoms on the R-plane of sapphire almost formed a square with sides of 4.75 and 5.12 Å along the [11$\bar{2}$0] and [$\bar{1}$101] directions, respectively. For the orientation of Au crystallites, the relationship Au (111) [1$\bar{1}$0] ‖ (1$\bar{1}$02) [$\bar{1}$101] Al_2_O_3_ was the most probable.

An R-II-type film was formed on the (111) Au surface without signs of anisotropy (Figure 1c,d). According to the AFM data, the RMS value of the surface roughness and the maximum height difference of the relief were 21 nm and 120 nm, respectively. According to XRD data, the ZnO film was [0001]-textured (Figure 2a). Since both structures had third-order symmetry, (0001)-oriented growth domains rotated by 180° would be formed in the growing ZnO film. In addition, weakly noticeable features were observed in the region of the 11$\bar{2}$0 reflection peak in the XRD pattern. When forming a ZnO film on (111) Au, there are almost ideal conditions for lattice conjugation due to the close values of $2•a_{\mathrm{Au}\left[1\bar{1}0\right]}$ and $a_{\mathrm{ZnO}\left[1\bar{1}00\right]}$. This explains the presence of [11$\bar{2}$0] ZnO crystallites in the film. The porous Au sublayer and the presence of [11$\bar{2}$0] ZnO crystallites slowed down the growth of [0001] ZnO crystallites, thereby smoothing the film surface. The size of the crystallites (Table 1) in the type-R-II film was increased in comparison with the type-R-I film, whereas there was an almost perfect correspondence of the parameter with the one from the Standard JCPDS pattern for ZnO (file no: 043-0002).

For comparison, ZnO films were synthesized on the C-plane of sapphire (type C films). Due to a significant discrepancy between the lattice parameters (up to 42%), the synthesis process on C-sapphire was close to the synthesis process on nonorienting substrates. A [0001]-textured film was formed that was azimuthally misoriented. As a result of the high nucleation density and geometric selection (at the expense of neighboring crystallites, those crystallites grow, the growth vector of which is oriented towards free space), a strongly developed surface was formed (Figure 1e,f). The RMS value of the roughness reached 40–50 nm, and the height difference of the relief was 400 nm.

A similar result was obtained in the case of the deposition of type-A films. The surface of the sample (Figure 1g) was a continuous layer, on which pronounced [0001] microcrystals up to 3 μm in height and width and elongated [10$\bar{1}$2] microcrystals were formed along the surface. The longitudinal and transverse dimensions of [10$\bar{1}$2] microcrystals were up to 8 μm and 1 μm, correspondingly, whereas their height was 1–2 μm. At the initial stage, in accordance with the classical concepts of the structural-geometric correspondence of lattices, a continuous (0001) ZnO film was formed. These results were confirmed by XRD data (Figure 2b, curve A). In addition to the main 0002 reflection peak, 10$\bar{1}$2 reflection and similar 10$\bar{1}$1 and 10$\bar{1}$3 reflections could be observed in the XRD patterns of the samples. Notably, all reflection peaks of ZnO films were shifted to the region of large angles in comparison with the data for the Standard JCPDS pattern for ZnO (file no: 043-0002), which indicates a lack of oxygen. In addition, the (0002) reflection was partially bifurcated: the type-A film contained crystallites with two values of the *c* parameter and different crystallite sizes (Table 1). It can be assumed that the parameter equal to 2.583 nm is characteristic of a continuous (0001) film with a smaller crystallite size, whereas the parameter equal to 2.589 nm is characteristic of individual [0001] microcrystals growing under conditions of low competition. A similar picture was also observed for [10$\bar{1}$2] ZnO microcrystals growing on the background of a continuous film (Table 1). The roughness of the continuous layer of type-A films, as in the case of type-C films, was high due to the growth of [0001] crystallites.

The morphologies (Figure 1h) and XRD patterns (Figure 2, curve M) observed for films of type-M differed from those observed for type-A films. The surface of the sample was a continuous layer, on which pronounced large [0001] microcrystals up to 10 µm high and up to 8 µm in diameter were formed. In addition to the main 0002 reflection peak, 10$\bar{1}1$, 10$\bar{1}$2, and 10$\bar{1}$3 reflections of relatively low intensity were also distinguished in the XRD patterns of the samples. In this case, the position and shape of the (0002) reflection for M-type and A-type films coincided with high accuracy and, as a result, the *c* parameter for both films coincided (Table 1). Therefore, in the case of the type-M film, one can also speak of a lack of oxygen; the differences were only in the size of the crystallites. Based on this, it can be assumed that a continuous film was formed due to crystallites of all four orientations (0001), (10$\bar{1}$1), (10$\bar{1}$2), and (10$\bar{1}$3), of which only [0001] crystallites grew at an early stage, subsequently becoming coarser due to crystallites of other orientations. In this case, the thickness of the continuous film did not increase; it retained its smoothness due to the small grain sizes. Thus, the formation of individual [0001]-microcrystals occurred not due to a change in the growth rate, but in accordance with the rule of geometric selection.

**Figure 3:**
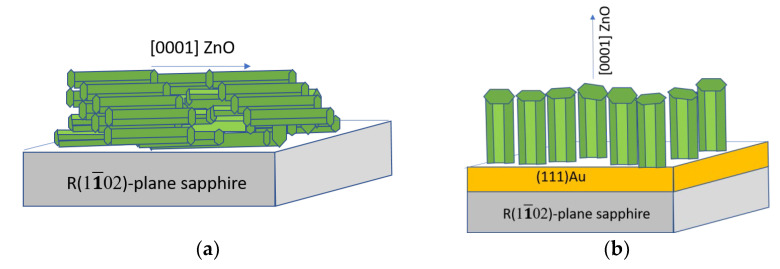

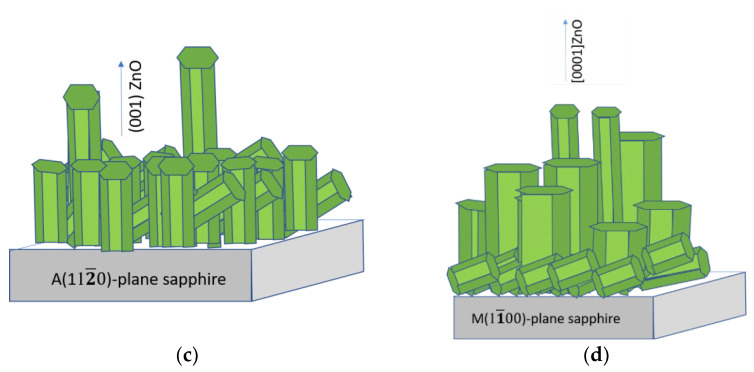
Proposed growth scheme for ZnO films of types R-I (**a**), R-II (**b**), A (**c**), and M (**d**).

The analysis performed in this study makes it possible to schematically (Figure 3) represent the growth of ZnO films and conditionally describe the features of their structure and morphology (Table 2). The results for A- and C-type films were similar, so only a description of the type-A film is presented in Table 2.

### 3.2. Optical and Luminescent Properties of Textured ZnO Films

#### 3.2.1. ZnO Films of Types R-I and R-II

**Figure 4:**
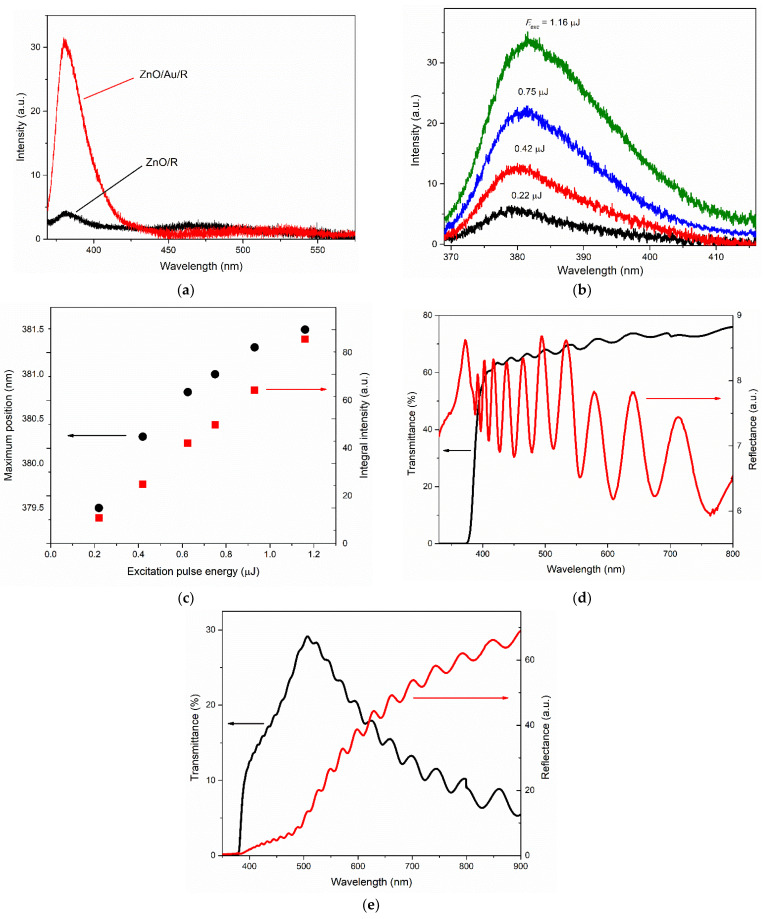
Optical and emission properties for the type-R-I and type-R-II films: (**a**) PL spectra of type-R-I film (black) and type-R-II film (red) recorded at excitation pulse energy *E_exc_* ≈ 0.4 µJ; (**b**) NBE emission spectra of type-R-II film at different *E_exc_*; (**c**) dependence of NBE emission integral intensity (red squares) and maximum position (black circles) on *E_exc_* for type-R-II film; (**d**,**e**) transmittance (black) and directional reflectance (red) spectra for type-R-I film (**d**) and type-R-II film (**e**).

Figure 4a shows the PL spectra of the ZnO films of types R-I and R-II registered in the near-UV and visible ranges at an excitation pulse energy of *E_exc_* ≈ 0.4 μJ. In both spectra, the near-UV band with a maximum at 381 nm and 380.3 nm for R-I- and R-II-type films, respectively, prevailed. This band is caused by ZnO near-band-edge (NBE) emission, and its intensity differs significantly between the samples. Emission of the samples in the visible region was observed in the wide range of 450–600 nm. However, its maximum position differed in the PL spectra of these two films: ~475 nm for the type-R-I film and ~510 nm for the type-R-II film. The ratio of the NBE and visible emission bands at a relatively low excitation level was ~6 and 1.5 in terms of integral intensities and ~25 and 2 in terms of maximum intensities for the two films, respectively. The ratio between the intensities of the NBE band between the films was ~8.

The evolution of the NBE emission spectra of the films with an increase in the photoexcitation intensity was demonstrated in the example of the type-R-II film (see Figure 4b) in view of the higher brightness of its emission. Figure 4c plots the dependences of the integral intensity *I_int_* of the NBE emission (integration range from 370 to 440 nm) and the position of its maximum on *E_exc_*. The character of the dependence, *I_int_* (*E_exc_*), is linear; the experimental points are satisfactorily approximated by straight lines. With an increase in the excitation intensity of more than five times, the maximum of the NBE emission band redshifts by ~2 nm (from 379.5 to 381.5 nm).

Significant differences between the samples were also observed in their reflectance and transmittance spectra, as shown in Figure 4d,e, respectively. The transmittance spectrum of the type-R-I sample (Figure 4d) was generally typical of ZnO films. It was characterized by a short-wave edge in the region of 377–379 nm, corresponding to the fundamental absorption edge of ZnO, and signal growth in the direction of long waves, demonstrating good transmittance in the visible region ~60–75%. The directional reflectance spectrum of the type-R-I sample showed strong oscillations associated with interference in the ZnO film. The local reflection maximum at 378 nm was, apparently, the superposition of the exciton resonance peak [35] and the interference pattern in this region.

The shape of the reflectance and transmittance spectra of the type-R-II sample (Figure 4e) was significantly affected by the presence of the gold layer, as well as interference in the ZnO film. Thus, the characteristic maximum in the region of ~507 nm in the transmittance spectrum (Figure 4e, black curve) was mainly determined by the mechanisms of light interaction with gold nanoislands. The appearance of this peak was the result of various processes: interband transitions from the filled *d*-bands to the conduction band on the short wavelength side and the growing absorption and reflection of light by free electrons on the long wavelength side [36]. The influence of strong absorption of the blue-violet part of the spectrum in the gold layer and significant reflection of light in the longer wavelength region of the visible range were clearly seen in the reflectance spectrum of the sample (Figure 4e, red curve). The position of the peak in the transmittance spectrum in our case was slightly affected by the interference pattern, as well as, to a lesser extent, by the spectrally nonuniform transmittance of the ZnO film, which increased towards long wavelengths and therefore slightly redshifted the peak. Note that a similar shape of the transmission spectrum has also been observed for very thin gold layers, down to 10–20 nm thick [37].

#### 3.2.2. Type-A ZnO Film

**Figure 5:**
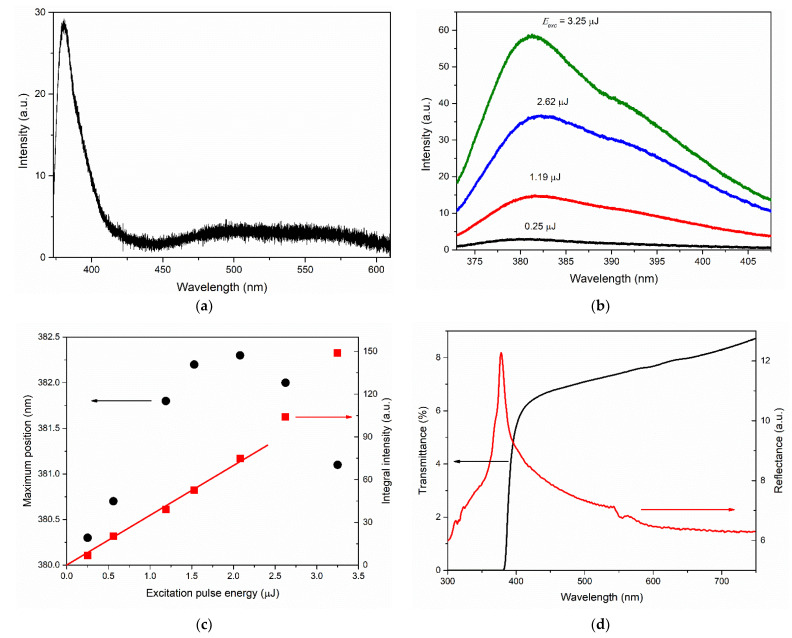
Optical and emission properties for the type-A film: (**a**) PL spectra recorded at *E_exc_* ≈ 0.25 µJ; (**b**) NBE emission spectra at different *E_exc_*; (**c**) dependence of NBE emission integral intensity (red squares) and maximum position (black circles) on *E_exc_*; (**d**) transmittance (black) and directional reflectance (red) spectra.

Figure 5a shows the PL spectrum of type-A film recorded at *E_exc_* ≈ 0.25 µJ. In this case, the NBE emission of the ZnO film is represented by a band with a maximum at 380.5 nm. Visible emission is represented by a wide band in the range of 450–600 nm. The ratio of the NBE and visible emission bands at this excitation level was ~1.5 in terms of integral intensities and ~9 in terms of maximum intensities.

The evolution of the NBE emission spectra of the type-A film with increasing *E_exc_* is shown in Figure 5b. Figure 5c shows the integrated intensity of the NBE emission *I_int_* and its maximum position vs. *E_exc_*. In this case, the *I_int_* (*E_exc_*) dependence was linear up to *E_exc_*~2 µJ, after which the slope of the dependence increased. Meanwhile, the maximum of the band was first redshifted by 2 nm and then blueshifted by 1.2 nm. In this case, the beginning of the blue shift corresponded to the beginning of a gradual increase in the slope of the *I_int_* (*E_exc_*) dependence. Moreover, as the excitation intensity increased, the FWHM of the NBE emission band first increased up to 25.1 nm and then decreased down to 22.3 nm.

In general, the type-A film was characterized by sufficiently bright UV radiation—about two times brighter than that of the type-R-II film—at the same excitation intensities (up to *E_exc_*~1 µJ).

The transmittance spectrum of the sample shown in Figure 5d (black curve) indicated a rather weak transmittance of the ZnO film in the visible region (6–8%). The transmittance edge corresponded to the region of 381–383 nm. The reflectance spectrum of the sample (Figure 5d, red curve) was characterized by an intense exciton resonance peak at 378 nm. The sample did not exhibit interference properties.

#### 3.2.3. Type-M ZnO Film

**Figure 6:**
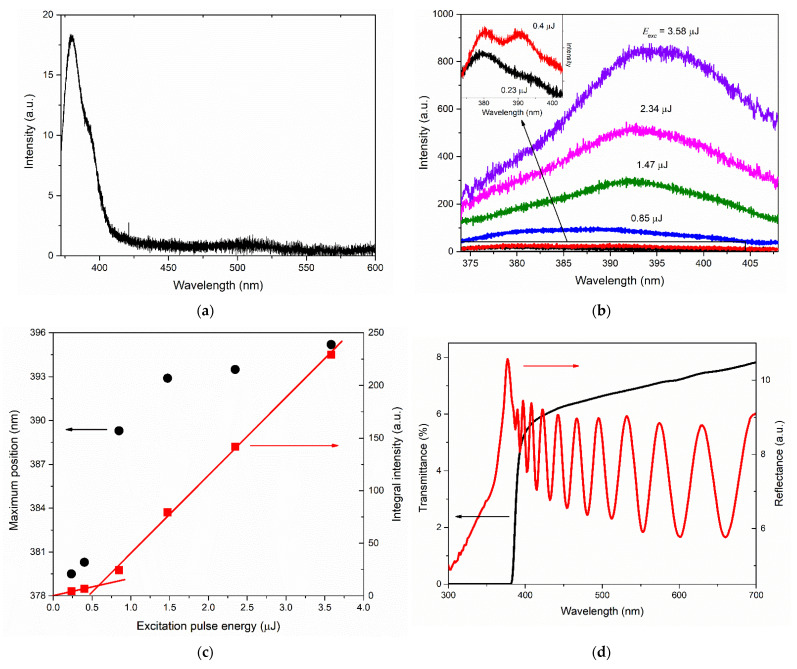
Optical and emission properties for the type-M film: (**a**) PL spectra recorded at *E_exc_* ≈ 0.25 µJ; (**b**) NBE emission spectra at different *E_exc_*; (**c**) dependence of NBE emission integral intensity (red squares) and maximum position (black circles) on *E_exc_*; (**d**) transmittance (black) and directional reflectance (red) spectra.

The PL spectrum in the UV and visible ranges of the type M ZnO film recorded at *E_exc_* ≈ 0.23 µJ is shown in Figure 6a. The ZnO NBE emission band in this case is located at 379.6 nm, and the visible radiation is represented by a small shoulder extending to approximately 550 nm. The ratio between the NBE and visible bands was ~3.5 in terms of integral intensities and ~22 in terms of maxima.

The evolution of the NBE emission spectra of the film with rising *E_exc_* ≈ 0.23 µJ shown in Figure 6b showed a significant transformation in the spectrum shape. Particularly, in the *E_exc_* range from 0.23 to 0.4 µJ, in parallel with the short-wavelength band that formed NBE emissions at lower excitation intensities, another longer-wavelength band with a maximum at 391 nm (at *E_exc_*~0.4 µJ) appeared and sharply increased in intensity. As *E_exc_* increased, this band grew much faster than the short-wavelength component, shifting to the long-wavelength side. The dependence of the integral intensity and the position of the spectrum maximum for this case are plotted in Figure 6c. In the *E_exc_* range from 0.4 to 0.8 μJ, the long-wavelength component began to predominate in the PL spectrum and determined its maximum. In the same range of *E_exc_*, the *I_int_* (*E_exc_*) dependence was characterized by a significant change in slope. Linear fitting of the sections before and after the slope change (see Figure 6c) gave the position of the bending at *E_exc_* ≈ 0.6 μJ.

Type-M film was characterized by almost the same level of transmittance as type-A film: 6–8% in the visible region (see Figure 6d, black curve). At the same time, type-M film demonstrated a rather contrasting interference pattern, which can be clearly seen in its reflectance spectrum (Figure 6d, red curve). The reflectance spectrum of the film also contained an intense exciton peak with a maximum at 377 nm, influenced by an interference pattern.

## 4. Discussion

The spectral position of the NBE emission of the studied films in the region of 379–381 nm, the absence of its strong shift, and its linear growth with an increase in the excitation intensity (for all films at relatively low excitation intensities) allow us to expect the participation of exciton mechanisms in the emission, provided that sufficiently low excitation intensities are used, at which the Mott threshold has not yet been exceeded.

In general, all the studied films, except the type-R-I film, demonstrated good UV luminescence intensity for bulk ZnO structures, comparable with other ZnO microcrystalline structures, for example, ceramics [38] and arrays of isometric microcrystals [39]. On the other hand, the NBE emission signal from the films was significantly lower as compared to that of ZnO nanocrystal arrays [40,41], which are significantly superior to films in terms of their surface-to-volume ratio, as well as ZnO microcrystals with optical modes [42,43]. In this case, films of types M and A approached the latter more significantly than the film of type R-II due to the superlinear response to photoexcitation.

At the same time, films of types A and M at low excitation intensities exhibited lower ratios of the NBE and visible emission bands. This ratio (according to the integral intensities) was almost two times lower than for the type-R-II film in the case of the type M film, and four times lower in the case of the type-A film. This presumably can be explained by the violation of stoichiometry in ZnO films. Type A and M films have a lattice parameter d_0001_ that is lower than that for the ZnO standard (Table 1). Meanwhile, it is frequently believed that ZnO lattice parameters change upon variation of the oxygen vacancy (V_o_) concentration. For example, in [44], the (002) peak shift in the XRD of the vacuum- and oxygen-annealed films was attributed to the V_o_ concentration variation, suggesting that the oxygen vacancies in the ZnO thin films are responsible for the decrease in the lattice parameters. On the other hand, however, a lack of oxygen, or oxygen non-stoichiometry, may result in a larger lattice parameter since a decrease in valency of the cation may increase its ionic radius. Despite the fact that the issue remains debatable, the deviation of the d_0001_ parameter from the standard one can be associated with a violation of stoichiometry. In this context, it is noteworthy that for the type-R-II film, for which the ratio of the NBE and visible emission bands was at the maximum, there was an ideal correspondence of the d_0001_ parameter with the ZnO standard one (Table 1). At the same time, in the case of the type-R-I film, for which the ratio of the NBE and visible emission bands was low, the $d_{11\bar{2}0}$ parameter, on the contrary, was larger than that for the ZnO standard. Thus, it turned out that for the films with parameters corresponding to the ZnO standard, the ratio of the NBE and visible emission bands was maximal.

Despite a good transmittance level, the type-R-I film showed the lowest UV luminescence intensity as compared to other ZnO films studied in this work. Defects located at the boundaries and near them can contribute to the nonradiative recombination of excitons, which generally reduces the yield the NBE emissions. In addition, this film was characterized by an excess of zinc, which could lead to the formation of interstitial Zn_i_ atoms and, as a consequence, to the appearance of additional centers of nonradiative recombination and an increase in the concentration of free electrons. The latter leads to screening of the electron-hole interaction and, as a result, to an additional decrease in the NBE emission intensity. Moreover, the presence of Zn_i_ can lead to emissions in the blue region [45]. This can explain the shorter wavelength maximum position of the visible emission band in the PL spectrum of this film compared to that observed for the type-R-II film, where visible emissions could be attributed mainly to the green luminescence of ZnO, commonly associated with oxygen vacancies. However, it should be noted that the low UV luminescence intensity of the type-R-I film was compensated for by contrast interference because of its low surface roughness.

Due to fundamental structural changes in the texture structure, the use of a porous gold buffer layer during the growth of ZnO on the R-plane of sapphire significantly increased the ZnO NBE emission intensity and the ratio of the UV and visible parts of the emission spectrum (from 1.5 in the case of the R-I-type film to 6 in the case of the type-R-II film). Such a growth method provided a significant smoothing of the surface of a growing *c*-axis oriented film and, as a result, retained the interference properties of a film, in contrast, for example, to a ZnO film growing on the A-plane of sapphire. It should be noted, however, that in this case the reflectance and transmittance spectra of such a structure were strongly affected by the use of a gold sublayer.

An increase in the growth rate of the NBE emission intensity with an increase in the photoexcitation intensity was observed in the case of films of types A and M. At the same time, the spectral behavior of NBE emissions differed significantly between these films. In particular, the shape of the UV PL spectrum of the type-M film at high excitation intensities distinguished this film strongly from all other samples. The two-band shape of the NBE emission spectrum, its threshold nature, and the rapid redshift of the long-wavelength band with increasing photoexcitation intensity indicated the appearance of stimulated emission (SE) in this film. In our recent work [46], we observed similar PL spectra, demonstrating the appearance of SE with a similar character and low threshold, in the case of polyhedral ZnO microcrystals supporting WGMs. At the same time, the type-M film differed from the other studied films with *c*-axis oriented crystallites due to the presence of large hexagonal [0001] microcrystallites on its surface. Taking into account the absence of SE of this type in other films, as well as the low SE threshold in the type-M film, we assume that SE arises in these hexagonal microcrystallites.

Taking into account the rather high photoexcitation intensities at which superlinearity appeared in the *I_int_* (*E_exc_*) dependence in the case of the type-A film, it can be assumed that this occurred in the presence of the electron–hole plasma (EHP). In this case, the blueshift of the NBE emission band observed at the same excitation intensities probably resulted from the dynamic Burstein–Moss effect (the band filling due to the intensive creation of electron-hole pairs via photoexcitation) [47,48]. The narrowing of the NBE emission band accompanying these effects indicated the onset of the EHP inversion and the appearance of SE in the film.

The reasons for such significant differences in the SE spectra of the type-A and type-M films have yet to be clarified, but the following can be preliminarily assumed. The [0001] microcrystals in the type-A film were smaller and had a much lower degree of perfection (prismaticity) compared to similar crystals in the type-M film (see Figure 2g,h). This probably hindered SE excitation in such microcrystallites, since they could not provide a significant optical path for light amplification, especially at rather low photoexcitation intensities, when exciton recombination mechanisms still existed. In this regard, SE in the case of the type-A film probably took place in a continuous layer of the film or, taking into account its rather large thickness, even more likely, in elongated [10$\bar{1}$2] microcrystals on the film’s surface, which distinguished this film from other films studied in this work. The length of the [10$\bar{1}$2] microcrystals can provide a single-pass optical gain (see [49], for example). However, the magnitude of such a gain is not as high as in microcavities. As a result, stimulation occurred only in the EHP regime. EHP formation in such microcrystals was also facilitated by their small (submicron) thickness (transverse size), which caused an increase in the density of electron-hole pairs in the volume of the crystals. This assumption needs to be verified in further studies.

Taking into account the sensitivity of the optical properties of microresonators, especially those with WGMs, to changes near their surface, one can expect sensing properties to be observed in these films as well. In this sense, further studies of ZnO films fabricated on the A- and M-planes of sapphire are relevant in order to elucidate the localization and nature of SE, to control the formation of individual microcrystals on the surface of the films, as well as to develop their possible applications.

The high brightness of NBE emissions of ZnO films grown on the A- and M-planes of sapphire was accompanied by their low transmission in the visible wavelength range and their longer-wavelength transmittance edge compared to the case of the type-R-I film. This was probably due to the greater intensity of light scattering in these films, which is presumably associated with the presence of [0001] microcrystals on their surface. At the same time, the type-M sample, in contrast to the type-A sample, exhibited an interference pattern in the reflection spectrum with a contrast level comparable with the interference in the type-R-I film. We attributed this phenomenon to the smoother surface of the continuous layer (in the regions between individual [0001] microcrystals) in the type-M film compared to the type-A film. Similar characteristics of the reflectance spectra between types-M and type-R-I films (amplitude, interference contrast, etc.) at significantly different transmittance values confirmed the assumptions made.

The high interference contrast observed in the case of type-R-I and type-M films promotes the use of such structures as optical coatings, i.e., antireflection coatings and refractive index sensors. In the latter case, attention should also be paid to the further development of films of the R-II type. The growth of ZnO on a gold nanolayer makes it possible to obtain a *c*-axis-oriented film with a significantly lower roughness compared to films grown on the A- and C-planes of sapphire. In addition, we believe that this technology will allow one to obtain interference films of much greater thickness (ten or more micrometers). In view of the much less narrow interference fringes, the efficiency of such films when used as sensitive elements of refractive index sensors increases significantly. In the simplest case of using one film in contact with the analyzed object, for example, a solution, the interference maximum with order *m,* is observed at the following wavelength [50]:

(4) $$\lambda_{m}=\frac{2d}{m+1/2}\sqrt[{}]{n_{\mathrm{ZnO}}^{2}-n_{s}{\left(C_{a}\right)}^{2}\sin\alpha^{2}},\;m=1,\;2,\;3,\ldots ,$$

where *d* is the thickness of the film with the refractive index $n_{\mathrm{ZnO}}$; $n_{s}\left(C_{a}\right)$ is the refractive index of the substance in contact with the film, which depends on the concentration of the analyte substance; and *α* is the angle of incidence/observation.

**Figure 7:**
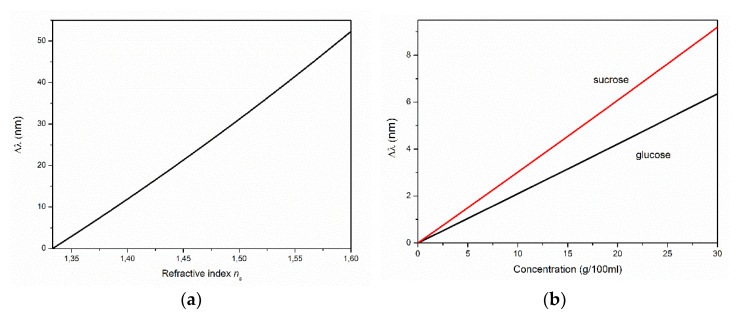
Estimation of the shift in the interference fringe with a maximum at 739 nm at *α* = 45° with an increase in (**a**) the refractive index of the substance in contact with the ZnO film; (**b**) the concentrations of glucose (black) and sucrose (red) in the aqueous solutions.

As an example, Figure 7 shows an estimate of the shift of one of the interference fringes with *m* = 13 at *α* = 45° with a change in $n_{s}$ (Figure 7a), as well as in the concentrations $C_{a}$ of glucose and sucrose in aqueous solutions in contact with the ZnO film with *d* = 3 µm (Figure 7b). For the estimates, we used the linear approximation $n_{s}\left(C_{a}\right)$ from [51] and the dispersion of $n_{\mathrm{ZnO}}\left(\lambda \right)$ from [52]. The sensitivity calculated from Figure 7 was ~190 nm/RIU. With sufficiently narrow interference bands (in the case of thick films), a shift of several nanometers can be easily tracked. The use of a narrow band source and/or two interference films can significantly increase the sensitivity of such a sensor. In this case, for example, films of types R-I, R-II, and/or M can be combined.

With a decrease in the thickness of a continuous gold nanofilm, its transmittance level increases and can reach a maximum of 10–20% at a thickness of 10–30 nm [53]. In our case, the relatively high transmittance (~30% in the maximum) seemed to be due to the island (porous) nature of the gold layer. From the perspective of using thinner gold layers, this would allow one to effectively use such a structure in reflected light, not only from the side of the ZnO film, but also from the side of the substrate, which is impossible when using opaque substrates, i.e., silicon. This geometry could be useful in the analysis of turbid solutions, which greatly attenuate the light passing through them.

**Figure 8:**
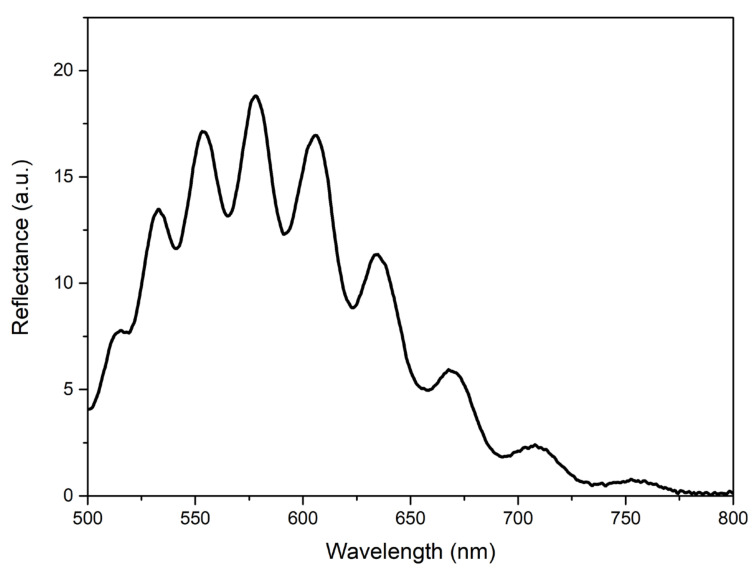
Reflectance spectrum of type-R-II film under illumination with a conventional commercial LED source under the angle of ~60°.

When developing an analyzer device, the developed structure (one or several) can be fixed, for example, in a standard spectrophotometer cuvette or, when working in flow mode, in a transparent tube. Unlike many sensors, such as waveguide-type ones, interference films do not require the use of laser radiation—it is sufficient to use a conventional LED. As an example, Figure 8 shows the reflectance spectrum obtained from the type-R-II film when illuminated with a conventional commercial LED.

The use of a high-resolution spectrometer is also an option. Moreover, when using a narrow-band LED as a light source, it is possible to use a conventional photodiode without using additional spectral devices. The fabrication technology of such sensors could also be simpler than that required for most sensors based on plasmonic materials, photonic crystals, etc.

**Figure 9:**
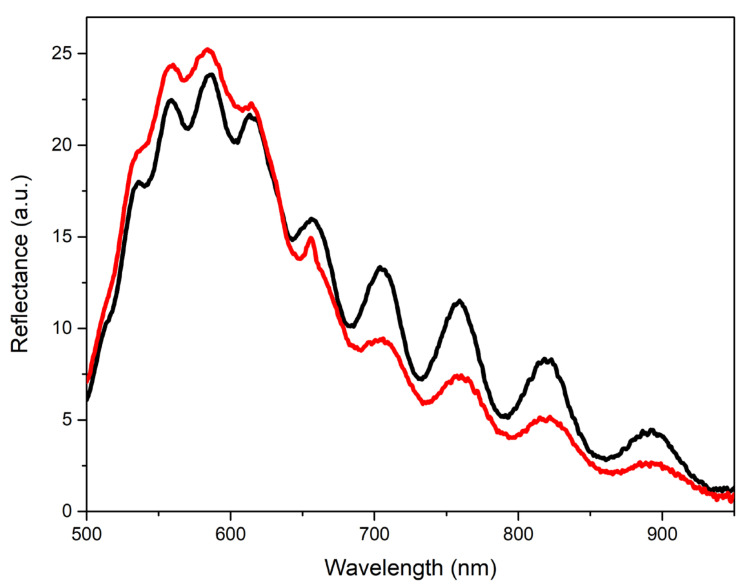
Reflectance spectra of a type-R-II film before (black) and after (red) covering it with a protecting Al_2_O_3_ nanolayer recorded under illumination with an LED source under the angle of ~60°.

Transparent coatings such as Al_2_O_3_ can be used to stabilize the ZnO film surface. Figure 9 shows the reflection spectra of one of the type-R-II films before and after the deposition of a protective Al_2_O_3_ layer under illumination by a LED source. In this case, the coating reduced the contrast of the interference fringes. However, work in this direction can also be continued.

## 5. Conclusions

We studied the synthesis processes, interference, and stimulated emission properties of ZnO film structures grown on R (1$\bar{1}$02)-, M (10$\bar{1}$0)-, A (11$\bar{2}$0)-, and C (0001)-planes of sapphire.

ZnO films grown on the R-plane of sapphire substrate were formed homogeneously, oriented by the (11$\bar{2}$0) plane parallel to the substrate. The R_q_ value and the height difference of the relief were 12 nm and 47 nm, respectively. In this case, the films had a small crystallite size of about 20 nm. When using a buffer layer of gold, the films were also formed homogeneously, but [0001]-texturing was observed. The RMS surface roughness and the height difference of the relief were 21 nm and 120 nm, respectively. Films grown on A-plane sapphire, as well as those grown on C-plane sapphire, exhibited high roughness values and grew with a (0001) plane parallel to the substrate. Moreover, the surfaces of the films were characterized by the presence of individual [0001] microcrystals up to 3 μm in height and [10$\bar{1}$2] microcrystals, elongated along the surface with longitudinal sizes up to 8 microns. When using an M-plane sapphire as a substrate, ZnO films grew morphologically inhomogeneously. Pronounced large [0001] microcrystals up to 10 µm high and up to 8 µm in diameter were formed in a continuous smooth layer. At the same time, the crystallinity of such films was the highest due to the perfection of the individual microcrystals.

The films grown on the A- and M-planes of sapphire were characterized by the highest brightness of UV radiation. In addition, these films demonstrated stimulated emission of various types. At the same time, in contrast to the ZnO film grown on A-plane sapphire, the film on M-plane sapphire exhibited noticeable interference properties. We assumed that [0001]-microcrystallites formed on the film surface were responsible for the appearance of stimulated emissions, and that this interference was realized in the film layer as a result of the low roughness of its surface in the regions between these microcrystals.

A study of the growth of ZnO films on a porous gold sublayer deposited on the R-plane sapphire substrate revealed differences in the spectral properties of films grown with and without gold. In particular, the use of gold resulted in a significant increase in the intensity of ZnO NBE emission compared to that of the film grown on a bare substrate. Both films exhibited interference properties; however, gold had a noticeable effect on the reflectance and transmittance spectra. In general, due to the significant smoothing of the *c*-axis-oriented film’s surface, we assumed that the use of gold can facilitate the fabrication of sufficiently thick (tens of micrometers) ZnO films with low surface roughness. The narrow interference fringes of such films will increase their resolution as elements of refractive-index sensors. Estimations of the sensitivity of the observed interference to changes in the refractive index of the surrounding medium—in particular, aqueous solutions of glucose and sucrose—suggested the possibility of using ZnO film structures in in situ studies of the photodegradation of such substances.

The results obtained emphasize the relevance of further studies of such ZnO film structures.

## Figures and Tables

**Table 1:** Parameters of the crystal structure of ZnO films.

Plane	Interplanar Distance, Å					Crystallite Size, nm
	Type R-I	Type R-II	Type A	Type M	Standard JCPDS Pattern for ZnO (File No: 043-0002)	
(11$\bar{2}$0)	1.633, 1.631	–	–	–	1.624	22.8, 20.2 (R-I)
(10$\bar{1}2$)	–	–	1.904, 1.899	–	1.910	78.9, 86.8 (A)
(0002)	–	2.603			2.603	61.2 (R-II)
			2.589, 2.583			75.6; 59.5 (A)
				2.589, 2.583		83.1; 138.6 (M)
	

**Table 2:** Comparative features of surface topography, morphology, and crystal structure of the fabricated ZnO films.

Film Type	Surface Topography	Morphology	Crystallinity
R-I	smooth (continuous layer)	homogeneous	low
R-II	smooth (continuous layer)	homogeneous	average
A	rough (continuous layer)	heterogeneous (continuous layer + individual microcrystals	high
M	smooth (continuous layer)	heterogeneous (continuous layer + individual microcrystals	highest

## Data Availability

Not applicable.
